# Biochemistry reference intervals for healthy elderly population in Asmara, Eritrea

**DOI:** 10.1186/s13104-017-3087-6

**Published:** 2017-12-19

**Authors:** Oliver Okoth Achila, Paulos Semere, Danait Andemichael, Harerta Gherezgihier, Senait Mehari, Adiam Amanuel, Tedalo Yohannes, Eyob Yohaness, Tzegezeab Goje

**Affiliations:** Department of Clinical Laboratory Services, Asmara College of Health Sciences (ACHS), P.O. Box 9540, Asmara, Eritrea

**Keywords:** Reference intervals, CLSI, Liver funtion, Electrolytes, Bilirubin, Lipids

## Abstract

**Objective:**

There is a scarcity of reference interval studies on the elderly in Africa. This study establishes reference interval for the elderly for some commonly used biochemical parameters. In this study, 255 conveniently sampled, healthy elderly, participants meeting Clinical and Laboratory Standards Institute (CLSI C28-A3) guidelines were enrolled. The results obtained may have utility in clinical diagnosis, patient management and research.

**Results:**

In general, the consensus reference interval established in this study tended to be higher than reference interval from Caucasian populations; but lower than those obtained from reference interval studies in several African countries. This pattern was observed in a number of analytes including Alanine aminotransferase; aspartate aminotransferase; alkaline phosphatase; sodium; potassium among others. Significant sex-related variations were also observed in total bilirubin; direct bilirubin; indirect bilirubin; albumin; sodium; chloride; plasma carbon dioxide, creatinine and anion gap. The results demonstrate that reference interval for Eritrean urban population differs from those derived from other African or North American populations. In this regard, the use of reference interval values obtained from Caucasian populations may result in misdiagnosis.

## Introduction

Clinical biochemistry reference intervals (RI) defined as the 2.5th and 97.5th percentiles of a healthy population’s distribution [1, 2]; have a wide range of application in patient care and management. This ranges from diagnosis of health disorders; evaluation of the toxicity of xenobiotics; disease staging and monitoring of treatment [3, 4].

To establish appropriate RI, the International Federation of Clinical Chemistry (IFCC) and Clinical and Laboratory Standards Institute (CLSI) recommend that RI should be derived locally [1]. The need to establish population-appropriate reference values is premised on a number of considerations. A prominent concern is the possible presence of substantial intra and inter-population variation that may influence laboratory RI. Underlying these differences are the observed variations in geography, diet, sex, climate, co-infections and genetics [4, 5].

These observations are in agreement with published data which have demonstrated the existence of significant inter-and-intra population differences in RI obtained within and between countries in specific biochemical analytes [4–8]. Often, the RI obtained from study cohorts in Africa differs significantly from those obtained from predominantly Caucasian cohorts in Europe and North America. Therefore, it has been suggested that using RI obtained elsewhere to a population of interest could potentially lead to misdiagnosis; inappropriate patient management and unnecessary use of resources [9].

Despite the established consensus that there are no universally applicable RI: most diagnostic laboratories in Africa continue to use RI obtained from textbooks, instruments manuals and reagents inserts; that have been derived predominantly from Caucasian populations in North America and Europe [4, 5, 7].

The observations highlighted above broadly describe the prevailing situation in Eritrea. To our knowledge, no age or sex -stratified studies have assessed clinical biochemistry RI in a controlled, systematic manner in any setting in the country for the elderly population. Therefore, this study is the first of its kind. In addition, it compares these findings to established RI from Massachusetts General Hospital (MGH), US and other published studies across Africa. In addition, it determines how many individuals would be misclassified as having adverse events (AE) according to the RI currently in use in the country.

## Main text

### Methods

#### Study population

In this cross-sectional study, conveniently sampled, healthy elderly subjects living in Asmara, the capital city of Eritrea, for at least 6 months were enrolled in from March 2016 through June 2016. Eligible participants, who consented to the study, were invited to the administrative center, where they were evaluated by a physician for evidence of acute and/or chronic illness. Based on clinical history, physical examination and CLSI–IFCC inclusion and exclusion criteria [2]; 255 individuals were ultimately selected to participate in the study. Briefly, the inclusion criteria included: male and female elders between 60 and 80 years old; residents of Asmara for ≥ 6 months; HCV and HBV sero-negative. Conversely, individuals with the following conditions were excluded: diabetes; cardiovascular disease; neoplasm (cancer/malignancies, liver disease, kidney disease, medication, transfusion or resent surgery; heavy smoking and alcohol consumption and significant recent illness.

#### Sample size

The CLSI–IFCC recommends that studies to establish reference intervals should have a minimum of 120 individuals in each grouping variable [2, 10]. In this study, population stratification was based on sex. In this regard, 255 (≥ 119 males and ≥ 131 females) were enrolled in the study.

#### Blood collection and serology

A maximum of 5 mL of whole blood was collected via venipuncture into BD Vacutainer test tubes (Becton–Dickinson Biosciences, San Jose, CA, USA). To minimize diurnal variation, all the samples were collected before noon (8.00–11.30 am). Samples were processed and analysed within 2 h. Samples that could not be analysed within 2 h were stored at 2–8 °C and processed within 3 days. Serological tests for hepatitis B virus (HBV) and hepatitis C virus (HCV) were undertaken using Determine™ HBsAg (Abbott Laboratories) and OraQuick^®^ HCV tests respectively.

#### Biochemical analysis

Biochemical analysis was performed using (Beckman Coulter: AU 480 Chemistry System) per the manufacturer’s instructions and Standard operating procedures (SOP). Each sample was analysed for aspartate aminotransferase (AST); alanine transferase (ALT); alkaline phosphatase (ALP); creatinine (Cre); blood urea nitrogen (BUN); total bilirubin (T-Bil); direct bilirubin; indirect bilirubin; total cholesterol (T-Chol); high density lipoprotein (HDL); sodium (Na); chloride (Cl); potassium (K); carbon dioxide (CO_2_) and albumin (Alb). anion gap was calculated from the following formula: anion gap = [Na^+^] + [K^+^] − [Cl^−^] + [HCO_3_^−^].

### Statistical analysis

All statistical analyses were carried out using SPSS Version 20 (SPSS version 20.0, SPSS Inc. Chicago, IL, USA). Determination of reference intervals was based on CLSI C28-A3 guidelines. Overall, sex-stratified data was assessed for Gaussian distribution using Kolmogorov–Smirnov test and Shapiro–Wilks test. Dixon method was used to identify outliers. The 2.5nd and 97.5th percentile, mean, median, range was determined. Depending on data distribution, the observed differences between males and females were evaluated using student *t* test or the Mann–Whitney U test. A two-sided P value of < 0.05 was considered significant. The percentages of participants with out of range (OOR) values were then calculated. The study consensus RI were subsequently compared to values currently in use by the Eritrean ministry of health—based on Beckman Coulter: AU 480 Chemistry System reagents inserts), specific African countries and US Massachusetts General Hospital (MGH).

#### Quality control

Normal and abnormal controls were run daily to monitor the accuracy of the biochemical analyser. The analyser was calibrated each day as per standards recommended by the manufacturer. External quality assessment was conducted at Sembel Hospital.

### Results

The mean; 95% confidence interval (CI) associated with the mean; median; range; 2.5th–97.5th percentile (RI); and P value (comparing means for males and females) for clinical biochemistry parameters are shown in Tables 1 and 2.

**Table 1:** The calculated population biochemical reference values for participants screened

Analyte	All	N	Mean	95% CI for mean	Median	Range	2.5th–97.5th percentile	P value (sex)
Aspartate aminotransferase (AST), IU/L	Combined	251	21.4	20.8–22.0	20.6	13–41	13.8–33.2	0.269
	Female	133	21.6	20.8–22.6	21.0	13–41	14.4–34.0	
	Male	118	21.0	20.2–21.8	21.0	12–33	13.0–32.0	
Alanine aminotransferase (ALT), IU/L	Combined	249	16.6	16.0–17.7	15.0	8–41	9.0–35.4	0.224
	Female	133	16.7	15.5–18.0	21.0	8–41	9–35.7	
	Male	116	17.0	15.8–18.2	16	8–29	8.0–29.0	
Alkaline phosphatase (ALP), IU/L	Combined	246	95.5	92.8–98.3	95.0	43–153	55.4–156.0	0.329
	Female	129	96.9	90.5–97.9	95.0	43–145	55–165	
	Male	117	94.2	92.8–101.1	95.0	52–153	51.7–153	
Creatinine, mg/dL	Combined	242	0.95	0.9–1.0	0.9	0.4–2.0	0.6–1.5	0.001
	Female	115	4.3	0.9–0.94	0.9	0.4–1.6	0.6–1.5	
	Male	127	4.4	1.0–1.05	1.0	0.7–2.0	0.7–1.9	
Blood urea nitrogen, mg/dL	Combined	245	13.5	13.0–14.0	13.0	6–41	7.1–22.0	0.388
	Male	116	13.5	12.6–14.4	16.0	6–41	7.0–26.5	
	Female	129	13.5	12.9–14.0	13	7–22	8.0–21.0	
Bilirubin (total), mg/dL	Combined	244	0.8	0.74–0.80	0.7	0.4–2.2	0.4–1.3	0.034
	Male	112	0.8	0.75–0.85	0.7	0.4–2.2	0.4–1.69	
	Female	126	0.7	0.7–0.77	0.7	0.4–1.5	0.4–1.20	
Bilirubin (direct), mg/dL	Combined	245	0.69	0.06–0.79	0.07	0.0–0.57	0.0–0.26	0.000
	Male	116	0.05	0.04–0.07	0.0	0.0–0.37	0.0–0.3	
	Female	129	0.08	0.07–0.1	0.09	0.0–0.57	0.0–0.25	
Bilirubin (indirect), mg/dL	Combined	245	0.16	0.12–0.20	0.00	0.0–1.9	0.0–1.0	0.000
	Male	116	0.03	0.0–0.06	0.00	0.0–1.9	0.0–0.66	
	Female	129	0.28	0.21–0.35	0.00	0.0–1.9	0.0–1.15

**Table 2:** The calculated population biochemical reference values for participants screened for the elderly study

Analyte	All	N	Mean	95% CI for mean	Median	Range	2.5th–97.5th percentile	P value (gender)
Total cholesterol (T-Chol) (mg/dL)	Combined	244	193	187–198	190.0	301–348	116–298	0.342
	Male	116	189.0	181–197	186.5	47–326	100–310	
	Female	128	196.0	188–203	192.0	118–348	130.0–296.0	
High density lipoprotein (HDL) (mg/dL)	Combined	244	50.0	48–51.2	48.0	30–133.0	31.1–80.0	0.605
	Male	115	50	47–52	48.0	30–133	41.0–80.0	
	Female	129	50.0	48–52	48.0	30–90.0	33.0–81.0	
Sodium (Na) (mmol/L)	Combined	245	140.5	140–141	140.0	131–156	135–147	0.000
	Male	116	140.0	139–140	139.0	131–149	135–145	
	Female	129	141.0	140–142	141.0	133–156	134–148	
Chloride (Cl) (mmol/L)	Combined	244	107	106.5–107	107	99–122	101–113	0.020
	Male	116	106	106–107	106	99.0–114	101–113	
	Female	128	107.4	107–108	107	99–122	100–113.5	
Potassium (K) (mmol/L)	Combined	245	4.3	4.3–4.4	4.30	3.3–5.90	3.61–5.30	0.426
	Male	116	4.4	4.27–4.4	4.30	3.3–5.70	3.7–5.51	
	Female	129	4.30	4.2–4.4	4.30	3.5–5.9	3.6–5.30	
Anion gap (μLU/mL)	Combined	235	8.2	4.1–9.0	9.0	− 24 to 25	7–21	0.000
	Male	108	5.7	4.1–7.3	8.0	0–17.0	0–15.6	
	Female	127	10.3	9.5–11.0	10.0	1.0–25.0	1.4–22.0	
Carbon dioxide (CO_2_) (mmol/L)	Combined	238	26.0	25–27	25.0	11–58.0	18–37.0	0.004
	Male	109	28.0	26–29	25.0	11.0–58.0	17.0–38.0	
	Female	129	24.0	23.7–24.8	24.0	16.0–34.0	18.2–32.5	
Albumin (Alb) (g/dL)	Combined	245	4.68	4.6–4.7	4.7	2.1–5.5	4–5.30	0.000
	Male	116	4.57	4.7–4.8	4.6	3.5–5.30	3.9–5.2	
	Female	129	4.80	4.7–4.8	4.8	3.4–5.5	4.1–5.5	

According to the data, there was no significant difference between males and females in the levels of AST (13.0–32.0 IU/L versus 14.4–34.0 IU/L; P = 0.269); ALT (8.0–29.0 IU/L versus 9–35.7; P = 0.224); ALP (51.7–153 IU/L versus 55–165 IU/L; P = 0. 0.329) and BUN (7.0–26.5 IU/L versus 8.0–21.0 IU/L; P = 0.388).

However, males had significantly higher values in the levels of Creatinine (0.7–1.9 mg/dL versus 0.6–1.5 mg/dL; P = 0.001); Total bilirubin (T-Bil) (0.4–1.69 mg/dL versus 0.4–1.20 mg/dL; P = 0.034) and direct bilirubin (DBil) (0.0–0.3 mg/dL versus 0.0–0.25 mg/dL; P = 0.000) compared to females. Conversely, females had significantly higher levels of indirect bilirubin (0.0–1.15 mg/dL versus 0.00–0.66 mg/dL; P = 0.000) compared to males.

Further, there was no significant variability between males and females in T-Chol (100–310 mg/dL versus 130–296.0 mg/dL; P = 0.342); HDL (41–80.0 mg/dL versus 33.0–80.0 mg/dL; P = 0.605) and levels of potassium (3.61–5.51 mmol/L versus 3.6–5.30 mmol/L; P = 0.426). Compared to their female counterparts, males had significantly higher levels of CO_2_ (17.0–55.2 g/dL versus 18.2–33.0 g/dL; P = 0.004). However, females had significantly higher levels of Na (134–148 mmol/L versus 135–145 mmol/L; P = 0.000); Cl (104–114 mmol/L versus 101–113 mmol/L; P = 0.02); Anion gap (1.4–22 µl/mL versus 0.0–15.6 µl/mL; P = 0.000) and Alb (4.1–5.5 g/dL versus 3.9–5.2 g/dL; P = 0.000) compared to males.

Table 3, lists the RI obtained in this study cohort, values recommended by the Eritrean ministry of health and US MGH RIs. The table’s out of range (OOR) percentage (%) column shows the proportion of participants whose results would have been graded as abnormal based on the current RI provided by the ministry. For additional comparisons, results from specific studies in Ghana [7]; Botswana [11]; US. MGH [12]; eastern and southern Africa [8] were used.

**Table 3:** Clinical biochemistry results, comparison intervals and out of range (OOR) values

Analyte	Units	Eritrea screened cohort	Eritrean Ministry of Health	Out of range (OOR)	Middle belt of Ghana [7]	Botswana TFD2 screened cohort [11]	US Massachusetts General Hospital [12]	Combined study (eastern and southern Africa [8])
Aspartate aminotransferase (ASP)	U/L	13.8–33.2	0–31	6.8	7–51	13.0–42.0	0–35	14–60
Alanine aminotransferase (ALT)	U/L	9–35.4	0–31	5.6	14–51	7.0–46.0	0–35	8–61
Alkaline phosphatase (ALP)	U/L	55.4–156	39–117	18.4	84–241	–	30–120	48–164
Creatinine (Cre)	mg/dL	0.6–1.5	0.5–1.1	15.5	–	0.5–1.1	0–1.33	–
Blood urea nitrogen (BUN)	mg/dL	7.1–22.0	6–20	6.2		5.0–21.0	10–20	–
Total bilirubin (TBIL)	mg/dL	0.4–1.3	0.0–1.0	12.0	0.3–2.6	0.2–1.8	0.3–1.0	0.2–2.2
Direct bilirubin (DBIL)	mg/dL	0.1–0.3	0.00–0.3	1.3	0.8–0.4	0.1–0.4	0.1–0.3	0.0–0.5
Total cholesterol (T-Chol)	mg/dL	50–357	0–200	25.0		–	–	–
High density lipoproteins (HDL)	mg/dL	31.0–80.0	40–100	22.5		–	–	–
Sodium (Na)	mmol/L	135–147	135–145	4.3	135–150	135–143	136–145	–
Chloride (Cl)	mmol/L	101–113	101–111	10.5	102–114	98.0–107	98–106	–
Potassium (K)	mmol/dL	3.63–5.30	3.6–5.0	5.4	3.6–5.2	3.6–5.2	3.5–5.0	–
Carbon dioxide (CO_2_)	mmol/L	18–37	21–31	29.5		–	21–30	–
Albumin (ALB)	g/dL	4–5.30	3.4–4.8	30.6	3.3–5.0	–	–	–

*OOR* out of order range

### Discussion

There is a scarcity of RI studies on the elderly in Africa. Existing publications on RI in Africa are generally limited to specific sub-populations: usually children or individuals ≤ 60 years. This situation is attributable to the fact that most studies are rationalised as critical adjuncts to clinical trials [4, 7, 8, 13, 14]. Therefore, verification of RI used in clinical laboratories or establishment of new RI for the elderly has not been a priority. Understandably, some researchers have opined that establishing RI for geriatrics may have the inadvertent consequence of normalising age-related decline in physiological functions and the consequent sub-clinical diseases [15–17]. This, in their opinion, may desensitize clinicians on specific mortality risks of ageing. However, difference in the levels of specific analytes between elderly and youthful persons does not mean some pathology is present. Aware of the latter assertion, several studies on elderly RI have been published [15, 18].

Key findings include the fact that the RI estimated in this study for several analytes were higher than those derived from US MGH and reagent inserts but lower; significantly, at times, than those from previous studies in other African countries [3–5, 7, 8, 13]. Liver function test demonstrated this pattern (Table 3). The unusual nature of this observation is compounded by the fact that the cohorts in the studies from other African countries are generally younger. The results are also in agreement with the RI proposed by Pathology Harmony, a UK-based project which was initiated to harmonize RI for common analytes across UK [19]. However, the findings suggest that the current RI used in Eritrea for common liver function test (LFT) may result in patient misclassification.

The data also demonstrated that TBIL was comparable to those obtained in some studies in Africa. The upper limit for TBil was twice as high as those recommended by Eritrean Ministry of Health but similar to that obtained in the combined Eastern and South African study [8]. The etiology of high TBIL may be linked to hemolysis, malnutrition or physical exertion. However, some researchers have suggested that the relatively high TBIL observed across Africa may be related to common environmental or genetic factor [7]. The upper limit for DBIL was comparable to US MGH intervals but lower than those from other studies in Africa. A sex-related difference was observed all bilirubin indices (Table 1). At present, the medical significant of these differences remains unclear.

The RI for creatinine and BUN in this study were slightly higher than those obtained in the other studies (Table 3) with Cre demonstrating a sex-related difference. These findings are consistent with established pathophysiological orthodoxy which acknowledges age and sex—related differences in Cre and BUN [20].

We also observed slightly higher upper limit levels for electrolytes. A sex related difference was also observed in the mean values of Na^+^ and Cl^−^ will females having slightly higher values of Na^+^ and Cl^−^. However, there was no sex-related difference in the average value of K^+^. The RI obtained for Na^+^, K^+^ and Cl^−^ in this study are slightly higher than consensus measurements from a study in elderly population in Australia [15]. Similarly, the upper limit for K^+^ was slightly higher when compared to the study from Ghana [7] but significantly lower when compared to the study from Nigeria [21]. However, it was similar to UK pathology Harmony consensus RI [19]. In general, the extracellular concentration of Na^+^ and K^+^ depend of a range of factors including PH, kidney function, diet, diuretics use and haemolysis, among others [20, 22]. Therefore, the slight rise in K^+^ maybe related to age-related degeneration in lung and renal function.

In this study, the upper limit for plasma RI for CO_2_ was significantly higher when compared to the RI currently in use. A significant sex-related variation was also observed in plasma concentration of CO_2_. The elevated level of CO_2_ in men may be related to a history of smoking.

### Conclusions

In agreement with some previous publications, this study demonstrated the existence of inter-country variations in several analytes. In general, the consensus RI established in this study tended to be higher than RI from Caucasian populations; but lower than those obtained from RI studies in several African countries. Significant sex-specific variations were also observed. In some instances (DBil, Cl^−^, Alb), the absolute values of the observed differences were small. However, substantial differences warranting further studies or the establishment of sex specific RI for some indices (plasma CO_2_, Na^+^, Anion Gap and Cre) exists.

### Limitations

The study population comprised of self-selected, urban—dwelling population hence the applicability of the established RI for rural population’s remains unclear. In addition, we did not screen for all medical conditions, associated with the elderly, which might have impacted on specific measurements. Participants with subclinical conditions or parasitic infections may have been recruited inadvertently. In addition, information on a range of factors which may influence specific analytes like diet, history of smoking/and or alcoholism, among others, was not collected. Despite the highlighted limitations, the RI obtained in this study should provide valuable guidelines for clinical management of patients and interpretation of clinical research data.

## Abbreviations

AE: adverse events
Alb: albumin
ALP: alkaline phosphatase
ALT: alanine transferase
AST: aspartate aminotransferase
BUN: blood urea nitrogen
Cl: chloride
CLSI: Clinical and Laboratory Standards Institute
CO_2_: carbon dioxide
Cre: creatinine
DBIL: direct bilirubin
HBsAg: hepatitis B surface antigen
HBV: hepatitis B virus
HCO_3_: bicarbonate
HCV: hepatitis C virus
HDL: high density lipoprotein
IFCC: International Federation of Clinical Chemistry
K: potassium
MGH: Massachisetts General Hospital
Na: sodium
OOR: out of order
RI: reference range
SOP: standard operating procedures
T-Bil: total bilirubin
T-Chol: total cholesterol

## References

[1] National Committee on Clinical Laboratory Standards (NCCLS) (2000). How to define and determine reference intervals in the clinical laboratory; approved guideline.

[2] Clinical and Laboratory Standards Institute (CLSI) (2008). Defining, establishing, and verifying reference intervals in the clinical laboratory; approved guideline.

[3] Buchanan AM, Muro FJ, Gratz J, Crumo JA, Musyoka AM, Sichangi MW, Morrisey AB (2010). Establishment of haematological and immunological reference values for healthy Tanzanian children in Kilimanjaro Region. Trop Med Int Health.

[4] Odhiambo C, Oyaro B, Odipo R, Otieno F, Alemnji G, Williamson J (2015). Evaluation of locally established reference intervals for hematology and biochemistry parameters in western Kenya. PLoS ONE.

[5] Kibaya RS, Bautista CT, Sawe FK, Shaffer DN, Sateren WB (2008). Reference ranges for the clinical laboratory derived from a rural population in Kericho, Kenya. PLoS ONE.

[6] Saathoff E, Schneider P, Kleinfeldt V, Geis S, Haule D (2008). Laboratory reference values for healthy adults from southern Tanzania. Trop Med Int Health.

[7] Dosoo DK, Kayan K, Adu-Gyasi D, Kwara E, Ocran J (2012). Haematological and biochemical reference values for healthy adults in the middle belt of Ghana. PLoS ONE.

[8] Karita E, Ketter N, Price MA, Kayitenkore K, Kaleebu P (2009). CLSI derived hematology and biochemistry reference intervals for healthy adults in eastern and southern Africa. PLoS ONE.

[9] Zeh C, Amornkul PN, Inzaule S, Ondoa P, Oyaro B (2011). Population-based biochemistry, immunologic and hematological reference values for adolescents and young adults in a rural population in western Kenya. PLoS ONE.

[10] Solberg HE (1987). International Federation of Clinical Chemistry (IFCC), Scientific Committee, Clinical Section, Expert Panel on Theory of Reference Values, and International Committee for Standardization in Haematology (ICSH), Standing Committee on Reference Values. Approved Recommendation (1986) on the theory of reference values. Part 1. The concept of reference values. J Clin Chem Clin Biochem.

[11] Segolodi TM, Henderson FL, Rose CE, Turner KT, Zeh C (2014). Normal laboratory reference intervals among healthy adults screened for a HIV pre-exposure prophylaxis clinical trial in Botswana. PLoS ONE.

[12] Kratz A, Ferraro M, Sluss PM, Lewandrowski KB (2004). Case records of the Massachusetts General Hospital. Weekly clinicopathological exercises. Laboratory reference values. N Engl J Med.

[13] Eller LA, Eller MA, Ouma B, Kataaha P, Kyabaggu D (2008). Reference intervals in healthy adult Ugandan blood donors and their impact on conducting international vaccine trials. PLoS ONE.

[14] Tembe N, Joaquim O, Alfai E, Sitoe N, Viegas E (2014). Reference values for clinical laboratory parameters in young adults in Maputo, Mozambique. PLoS ONE.

[15] Janu MR, Creasey H, Grayson DA, Cullen JS, Whyte S, Brooks WS, Waite LM, Broe GA (2003). Laboratory results in the elderly: the Sydney Older Persons Study. Ann Clin Biochem.

[16] Pokorski RJ (1990). Laboratory values in the elderly. J Insur Med.

[17] Edvardsson M, Levander MS, Ernerudh J, Theodorsson E, Grodzinsky E (2015). Clinical use of conventional reference intervals in the frail elderly. J Eval Clin Pract.

[18] Zhang GM, Xia YJ, Guo XX, Zhu BL, Zhang GM, Ma XB, Yu H, Wang HJ, Wang GS, Yang L, Zhou YT (2014). Reference intervals of total bilirubin, ALT, AST, and creatinine in healthy elderly Chinese. Med Sci Monit.

[19] Berg J, Lane V (2011). Pathology harmony; a pragmatic and scientific approach to unfounded variation in the clinical laboratory. Ann Clin Biochem.

[20] Carreiro-Lewandowski E, Bishop M, Fody E, Schoeff L (2013). Basic principles and practices. Clinical chemistry, techniques, principles and correlations.

[21] Miri-Dashe T, Osawe S, Tokdung M, Daniel N, Choji RP, Mamman I (2014). Comprehensive reference ranges for hematology and clinical chemistry laboratory parameters derived from normal Nigerian adults. PLoS ONE.

[22] Sikaris KA (2014). Physiology and its importance for reference intervals. Clin Biochem Rev.

